# Public Health Legislation

**Published:** 1907-07-27

**Authors:** 


### Public Health and Hygiene.

### PUBLIC HEALTH LEGISLATION.

For many years it has been the custom of local authorities to add to the "sanitary" powers vested in them by the Public Health Act of 1875 by following the somewhat costly method of procedure which is offered by private bill legislation. The results have not been wholly satisfactory, and there is at present a lamentable lack of uniformity in the public health powers and duties of the various sanitary authorities. Mr. John Burns has it in mind, it is stated, to change all this by introducing next session, or as soon as may be, a consolidated Public Health Bill, in which will be included many clauses of a kind already obtained from Parliament in a variety of private Acts for different districts. In the meantime, however, and with a similar object in view, the Police and Sanitary Committee of the House of Commons, through its chairman, Mr. J. W. Wilson, M.P., has caused a Public Health Bill of considerable importance to be introduced. Mr. Wilson's Bill provided in the first place that local sanitary authorities might clothe themselves with the powers enumerated in the Bill by merely adopting the whole or any part thereof. At Mr. Burns's suggestion the Standing Committee has now altered this by making a Provisional Order of the Local Government Board a necessary preliminary to the acquisition of the powers of the Bill.

### The Public Health Bill.

Many other clauses, notably those dealing with milk from tuberculous cows, the regulation of the ice-cream trade, and the "original vendors" of unsound food, have been eliminated in view of Mr. Burns's promise to tackle these questions in another way at an early date. But the sanitation clauses that remain are still of much value, and it is to be hoped that expectations of a rapid and unopposed passage of the measure through Parliament may be realised. Some twenty clauses, for example, deal with infectious diseases, especially in relation to the conveyance of infection in milk, in clothes at the laundry, in public-library books, and in school children. Dairymen, laundrymen, and principals of schools may be required, when infection is suspected, by local sanitary authorities to furnish lists of sources of milk supply, laundry customers, and scholars respectively. Medical officers of health may be authorised to examine school children and to exclude "for such period as he shall consider requisite any scholar who, in his opinion, is suffering from infectious disease."

Three clauses, dealing with public ambulances, and the cost of hospital and nursing treatment, deserve to be quoted *verbatim*:—

Clause 54.—"The local authority may provide and maintain an ambulance for use in any case of accident, or other sudden or urgent disability, together with suitable attendants, and means of traction and other requisites; and may allow the ambulance to be used by any other local authority or person subject to such terms and conditions as may be agreed upon."

Clause 66.—"Nothing in Section 132 of the Public Health Act, 1875, with respect to the recovery of the cost of maintenance in a hospital shall require the local authority to recover the cost of maintenance from a patient who is not a pauper where the local authorities have satisfied themselves that the circumstances of the case are such as to justify the remission of the debt."

Clause 73.—(1) "The local authority may provide nurses for attendance on patients suffering from any infectious disease in their district who, owing to want of accommodation at the hospital or danger of infection, cannot be removed to the hospital, or in cases where removal to the hospital is likely to endanger the patient's health.

(2) "The local authority may charge such reasonable sums for the services of nurses provided by them as they may think fit.

(3) "Nothing in this section shall be deemed to take away or diminish the necessity for providing proper hospital accommodation for persons suffering from infectious disease."

The wording of these clauses is of importance as showing the exact extent to which the Legislature and the Local Government Board are prepared at present to confer these important powers upon sanitary authorities.

One part of the Bill is almost wholly devoted to such matters as the provision of proper and sufficient sanitary conveniences in the public streets, in public houses and in dwelling houses. Of these clauses it may be said that they are well calculated to give all necessary powers in these matters to those local authorities who have the clauses applied by the Local Government Board's Provisional Order. Lodging-houses, parks, sky signs, slaughter-houses, marine-store dealers and bathing places are among the remaining subjects dealt with, the clauses being for the most part of a type quite familiar to those who have followed the "health" legislation by private Bills during recent years.

In the meanwhile the London County Council has taken preliminary steps with a view to legislation next session for the better sanitary protection of the Metropolis. The Council has decided to consider detailed proposals, to be submitted later to Parliament, with a view of conferring on the Metropolitan Borough Councils, which are the executive sanitary authorities in London, powers to require the surrender, for the purpose of destruction, of unsound food, to regulate the conditions prevailing at places where food is sold or prepared for sale, and to regulate the businesses of fried fish dealers and fish curers. These proposals are to be submitted in detail and at an early date by the Public Health Committee of the London County Council, on which no fewer than five medical men serve. Apart altogether from the sensational discoveries of the lay Press, there can be little doubt that temperate legislation, inspired by sound technical knowledge and backed by medical opinion, is needed in the interests of the poorer classes of London's teeming population; and it would be well if all local sanitary authorities were able to number a substantial proportion of medical practitioners among their aldermen and councillors. On all such bodies there is scope for the activity of the medical profession.

